# Association between *ESRα* and *ESRβ* polymorphisms and prostate cancer risk: meta-analysis

**DOI:** 10.3389/fonc.2025.1630363

**Published:** 2025-12-08

**Authors:** Xiaohui Bai, Li Gao, Jiawei Zhao, Wentao Liao, Yujing Chen, Xiaotong Guo, Jiamin Jin, Bo Ge

**Affiliations:** 1 Department of Urology, The Second Affiliated Hospital of Guilin Medical University, Guilin, Guangxi, China; 2 Key Laboratory of Tumor Immunology and Microenvironmental Regulation, Guilin Medical University, Guilin, Guangxi, China; 3 Guangxi Health Commission Key Laboratory of Tumor Immunology and Receptor-Targeted Drug Basic Research, Guilin Medical University, Guilin, Guangxi, China

**Keywords:** *ESR*, polymorphism, prostate cancer, meta-analysis, FPRP, BFDP

## Abstract

**Background:**

Gene polymorphisms of *ESRα Pvull*(rs2234693), *Xbal*(rs9340799), and *ESRβ Alul*(rs4986938) and *RsaI* (rs1256049), have been investigated for their associations with prostate cancer risk. However, the nature of these relationships remains ambiguous. Therefore, the present study aimed to further clarify the association between *ESR* gene polymorphisms *and* prostate cancer.

**Objective:**

To investigate the association between *ESRα Pvull*(rs2234693), *Xbal*(rs9340799), and *ESRβ Alul*(rs4986938), *Rsal*(rs1256049) polymorphisms and prostate cancer risk.

**Materials and methods:**

PubMed, Medline, and CNKI were searched. Associations were assessed using odds ratios (ORs) with 95% confidence intervals (CIs). The false-positive report probability (FPRP), Bayesian false discovery probability (BFDP), and Venetian criteria were used to evaluate the credibility of statistically significant findings.

**Results:**

We found for the first time that, overall, the *ESRα PvuII* polymorphism was significantly associated with a reduced risk of prostate cancer (pp *vs.* Pp + PP: OR = 0.83, 95% CI = 0.71–0.97; pp *vs.* PP: OR = 0.75, 95% CI = 0.57–0.99; p *vs.* P: OR = 0.88, 95% CI = 0.78–0.99). A similarly reduced risk was observed in Caucasians (pp + Pp *vs.* PP: OR = 0.01, 95% CI = 0.01–0.04). By contrast, the **ESRα***PvuII* polymorphism increased prostate cancer risk among Africans (pp + Pp *vs.* PP: OR = 2.38, 95% CI = 1.61–3.51). For *ESRβ RsaI*, we observed a reduced risk of prostate cancer in Asians (r *vs.* R: OR = 0.87, 95% CI = 0.77–0.98). However, no significant associations were identified for *ESRα XbaI* or *ESRβ AluI*. When evaluating credibility using the FPRP, BFDP, and Venetian criteria, no statistically robust associations were confirmed.

**Conclusions:**

Overall, the results suggest a potential association between the *ESRα PvuII* and *ESRβ RsaI* polymorphisms and prostate cancer risk, although the credibility assessments did not support statistically robust relationships.

## Introduction

Prostate cancer (PCa) is a highly prevalent malignancy among men worldwide, particularly in Western countries, where it remains a leading cause of cancer-related mortality and a major public health concern (1). Although the incidence of PCa in Asian populations has historically been lower than in Western nations, epidemiological trends indicate a steady rise in cases, attributed to factors such as socioeconomic development, demographic aging, adoption of Westernized dietary habits, and lifestyle changes (2). Despite advances in early detection and therapeutic interventions, the precise etiology and molecular mechanisms underlying PCa pathogenesis remain incompletely understood. Current evidence suggests that both genetic susceptibility and environmental influences contribute to PCa development, with gene polymorphisms serving as an important biological determinant of inherited risk (3).

Estrogen receptors (ESRs) are nuclear macromolecules that mediate the biological effects of estrogen (4). Two isoforms exist—ESRα and ESRβ—which differ in the C-terminal ligand-binding region and the N-terminal activation domain (5). The *ESRα* gene consists of eight exons spanning approximately 140 kb on chromosome 6q25.1, whereas the *ESRβ* gene consists of eight exons spanning approximately 40 kb on chromosome 14q23–24.1 (6). Studies have shown that *ESRα* may act as an oncogenic factor that promotes tumor cell proliferation, while *ESRβ* is considered protective, exhibiting anti-cancer and pro-apoptotic effects. Genetic polymorphisms in the *ESRα* and ESRβ genes may lead to altered transcription or affect transcript stability (7). Therefore, polymorphisms in ESRα and ESRβ may represent potential risk factors for prostate cancer.

To date, numerous studies have examined the associations between *ESRα and ESRβ* gene polymorphisms and prostate cancer risk (8–15, 18–41). However, the evidence remains inconsistent, with some reports identifying significant associations while others report null findings. Eight meta-analyses have evaluated these associations, yet their conclusions remain conflicting (8–15). In addition, no previous meta-analysis performed a credibility assessment of statistically significant findings. Therefore, the present study further investigated the association between ESRα and ESRβ gene polymorphisms and prostate cancer risk.

## Materials and methods

PRISMA guidelines were followed in this meta-analysis (42).

### Search strategy

Articles were retrieved from PubMed, Medline, and CNKI. The search strategy was as follows: (“estrogen receptors” OR “ESR” OR “estrogen receptor alpha” OR “estrogen receptor beta” OR “ER alpha” OR “ER beta” OR “estrogen receptor 1” OR “estrogen receptor 2” OR “ESR1” OR “ESR2”) AND (“polymorphism” OR “gene” OR “SNP”) AND (“prostate cancer” OR “PCa). Searches were conducted up until September 15, 2024. In addition, reference lists of previously published meta-analyses were carefully reviewed to identify all eligible studies (8–15).

### Selection criteria

Inclusion criteria were as follows: (i) case–control studies; (ii) studies that assessed associations between *ESRα PvuII* and *XbaI, and ESRβ AluI* and *RsaI* polymorphisms and prostate cancer risk; (iii) prostate cancer cases confirmed by histopathology. Exclusion criteria were as follows: (i) cross-sectional studies; (ii) case reports, reviews, letters, and meta-analyses; (iii) animal studies.

### Data extraction and quality assessment

The literature was screened by two independent reviewers, with disagreements resolved through discussion or by consultation with a third researcher. Extracted data included the first author, publication year, country, ethnicity, sample sizes of cases and controls, and genotype frequencies (**Supplementary Tables 2–5**). Study quality was independently evaluated by two authors using criteria reported in previous studies (16, 17), as presented in **Supplementary Table 1**. The quality assessment was based on a 15-point scale, and a score greater than 8 indicated a high-quality study.

### Statistical analysis

All statistical analyses were performed using STATA version 12.0. Five genetic models were evaluated (dominant, recessive, additive, over-dominant, and allele models). Hardy–Weinberg equilibrium (HWE) in the control group was assessed using the chi-square test (*P* < 0.05 was considered indicative of Hardy–Weinberg disequilibrium [HWD]) (43).

Heterogeneity among studies was assessed using the *Q* test and the I² statistic. Significant heterogeneity was defined as *P* < 0.10 and/or I² > 50% (44), in which case pooled ORs were calculated using a random-effects model; otherwise, a fixed-effects model was applied. Potential sources of heterogeneity were explored using meta-regression analysis (45). Subgroup analyses were conducted by ethnicity and by source of the control group. Sensitivity analyses were performed by including only studies of high quality and studies in HWE.

Egger’s test (46) and Begg’s test (47) were used to evaluate publication bias. The false-positive report probability (FPRP) (48), Bayesian false discovery probability (BFDP) (49), and Venetian criteria (50) were used to assess the credibility of statistically significant associations (*I*^2^ < 50%; FPRP < 0.2 and BFDP <0.8).

## Results

**Figure 1:**
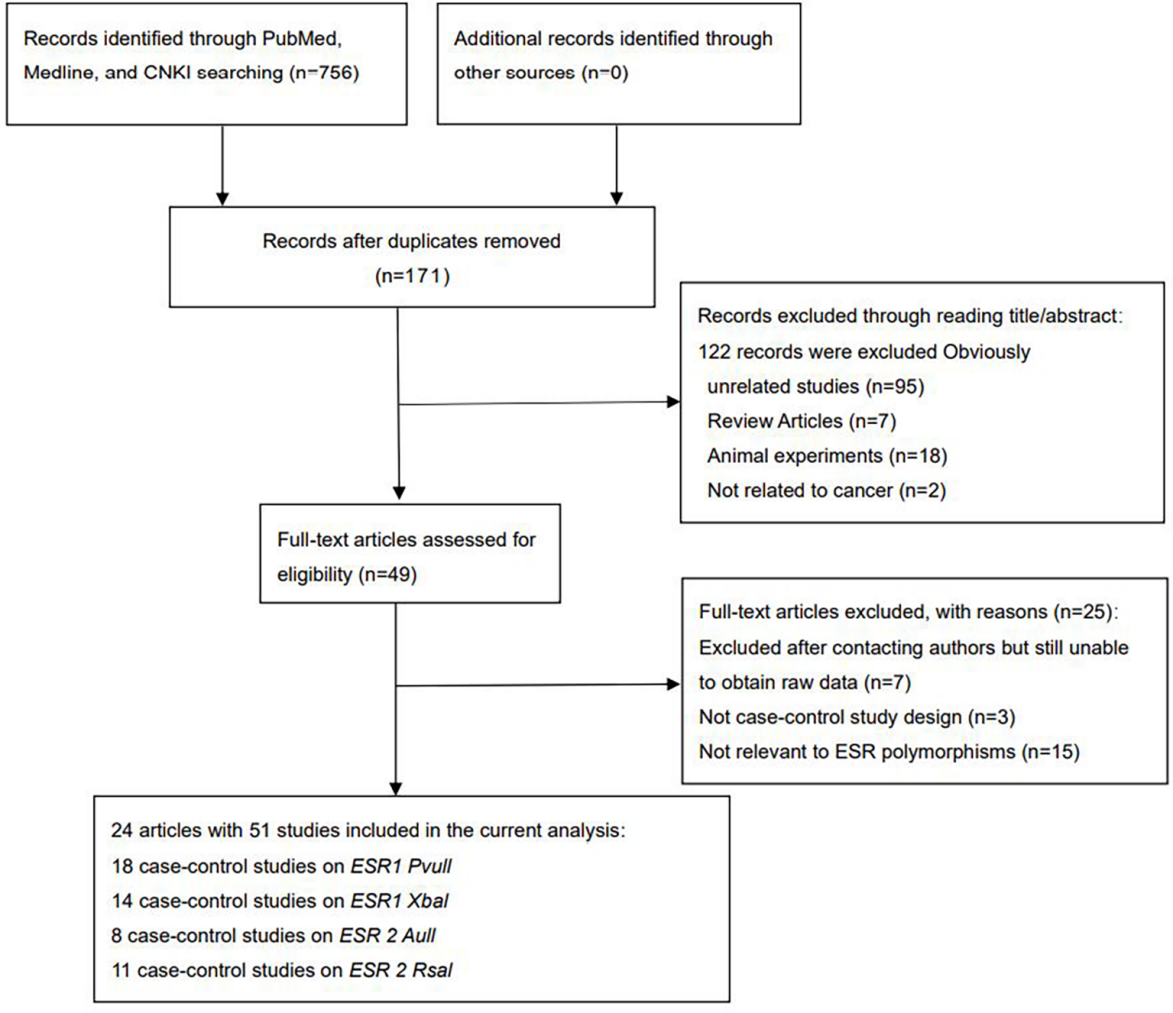
Flow diagram of the literature search.

Overall, 756 articles were retrieved (**Figure 1**). After screening titles and full texts, 24 articles comprising 51 studies were included (**Supplementary Tables 2–5**, **Figure 1**). A total of 26,454 cases and 33,941 controls were analyzed. As shown in **Supplementary Tables 2–5**, *ESRα Pvull* was reported in 18 studies (4,623 cases and 9,850 controls), *ESRα Xbal* in 14 studies (3,716 cases and 4,536 controls), *ESRβ Alul* in eight studies (8,674 cases and 9,498 controls), and *ESRβ Rsal* in 11 studies (9,390 cases and 10,057 controls). As detailed in **Supplementary Table 6**, the number of high-quality articles for each polymorphism was as follows: ESRα Pvull (n=10; with 8 low-quality), ESRα Xbal (n=5; with 9 low-quality), ESRβ Alul (n=3; with 5 low-quality), and ESRβ Rsal (n=7; with 4 low-quality).

### Quantitative synthesis

#### *ESRα Pvull* polymorphism

**Figure 2:**
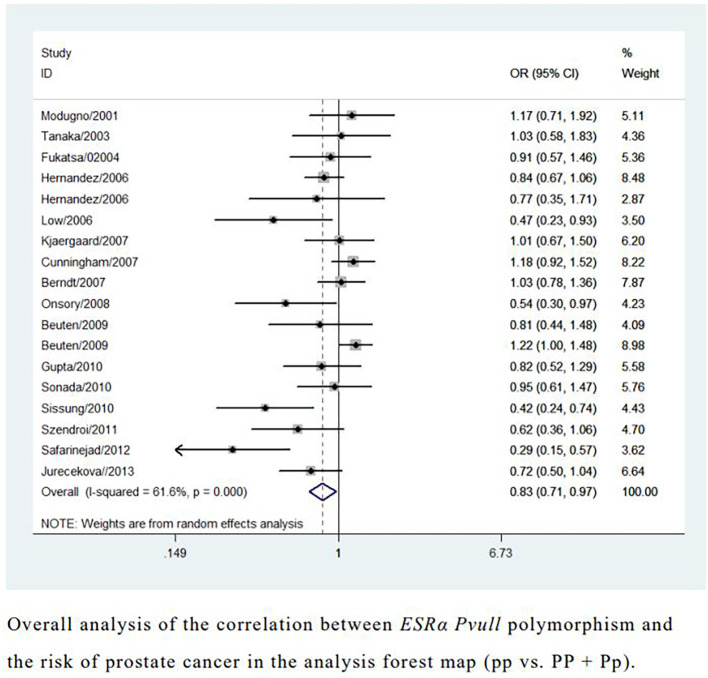
Overall analysis of the correlation between *ESRα Pvull* polymorphism and the risk of prostate cancer in the analysis forest map.

Overall analyses showed that the *ESRα* PvuII polymorphism was associated with a reduced risk of prostate cancer (pp *vs.* Pp + PP: OR = 0.83, 95% CI = 0.71–0.97; pp *vs.* PP: OR = 0.75, 95% CI = 0.57–0.99; p *vs.* P: OR = 0.88, 95% CI = 0.78–0.99; **Table 1** and **Figure 2**). A similarly reduced risk was observed in Caucasians (pp + Pp *vs.* PP: OR = 0.01, 95% CI = 0.01–0.04). However, the *ESRα* PvuII polymorphism was associated with an increased risk of prostate cancer in Africans (pp + Pp *vs.* PP: OR = 2.38, 95% CI = 1.61–3.51; **Table 1**). A reduced risk was also observed in hospital-based controls (pp *vs.* Pp + PP: OR = 0.80, 95% CI = 0.66–0.98; **Table 1**). Sensitivity analyses further confirmed the robustness of these findings.

**Table 1:** Meta-analysis of the association of *ESRα Pvull* polymorphism with risk of prostate cancer risk.

Variable	n (Cases/Controls)	pp + Pp *vs.* PP		pp *vs.* Pp + PP		pp *vs.* PP		pp + PP *vs*. Pp		p *vs.* P	
		OR (95% CI)	*P_h_/I^2^* (%)	OR (95% CI)	*P_h_/I^2^* (%)	OR (95% CI)	*P_h_/I^2^* (%)	OR (95% CI)	*P_h_/I^2^* (%)	OR (95% CI)	*P_h_/I^2^* (%)
Overall	18 (4623/9850)	1.35(0.83-2.21)	0.001/96.3	**0.83(0.71-0.97)**	0.001/61.6	**0.75(0.57-0.99)**	0.001/79.1	0.95(0.87-1.02)	0.163/24.7	**0.88(0.78-0.99)**	0.001/74.1
Ethnicity											
Caucasian	11 (3826/8543)	**0.01(0.01-0.04)**	0.541/20.7	0.81(0.66-1.01)	0.090/75	0.77(0.53-1.12)	0.324/86	0.93(0.81-1.06)	0.023/49.3	0.89(0.76-1.04)	0.056/82.4
Asian	5 (668/885)	0.97(0.58-1.64)	0.264/75.5	0.85(0.68-1.06)	0/0	0.73(0.50-1.06)	0.0534/29.5	0.99(0.81-1.21)	0/0	0.87(0.74-1.02)	0.004/13.3
African	2 (129/422)	**2.38(1.61-3.51)**	0/0	0.79(0.49-1.29)	0/0	0.66(0.37-1.16)	0/0	1.11(0.75-1.65)	0/0	0.81(0.61-1.07)	0/0
Source of controls											
HB	12 (3846/4935)	1.19(0.77-1.84)	0.535/93.9	**0.80(0.66-0.98)**	0.075/68.9	0.75(0.52-1.08)	0.329/84.7	0.92(0.84-1.01)	0.011/29.4	0.87(0.75-1.02)	0.056/80.8
PB	6 (777/4915)	1.75(0.43-7.17)	3.044/97.8	0.88(0.68-1.14)	0.043/42.3	0.75(0.55-1.01)	0.033/23	1.04(0.87-1.25)	0.006/10.4	0.88(0.77-1.01)	0.005/ 16.7
Sensitivity analysis											
HWE and Quality score > 8											
Overall	10 (2331/6076)	1.29(0.56-2.99)	0.001/97.5	**0.74(0.57-0.96)**	0.001/68.8	**0.70(0.51-0.97)**	0.002/66.1	0.94(0.83-1.06)	0.358/9.1	**0.86(0.74-0.98)**	0.007/60.5
Ethnicity											
Caucasian	7 (1920/5461)	1.25(0.40-3.95)	2.345/98.3	**0.65(0.45-0.94)**	0.183/78.4	**0.61(0.39-0.95)**	0.257/76.5	0.89(0.75-1.07)	0.020/36.4	**0.81(0.66-0.98)**	0.047/72.4
Asian	3 (411/615)	1.38(0.86-2.23)	0.109/61.5	0.95(0.72-1.26)	0/0	0.96(0.66-1.39)	0/0	0.98(0.76-1.26)	0/0	0.97(0.81-1.16)	0/0
Source of controls											
HB	6 (1792/1568)	0.87(0.54-1.40)	0.313/88.6	**0.68(0.46-0.99)**	0.169/76.9	0.65(0.40-1.05)	0.272/77.5	0.93(0.81-1.07)	0.012/25.9	0.83(0.68-1.02)	0.046/72.2
PB	4 (539/4508)	2.29(0.32-16.22)	3.930/98.4	0.82(0.56-1.20)	0.086/58.3	0.79(0.54-1.16)	0.048/31.5	0.96(0.77-1.18)	0.002/3.9	0.89(0.73-1.08)	0.015/37.7
Egger’s test											
*P_E_*		0.865		0.586		0.961		0.694		0.72	

*ESRα Pvull:* allele model: p vs. P, additive model: pp vs. PP, dominant model: pp + Pp vs. PP, recessive model: pp vs. PP + Pp over-dominant model: pp + PP vs. Pp; HB: hospital-based; PB: population-based. Bold values highlight the data with significant correlations.

#### *ESRβ Rsal* polymorphism

**Figure 3:**
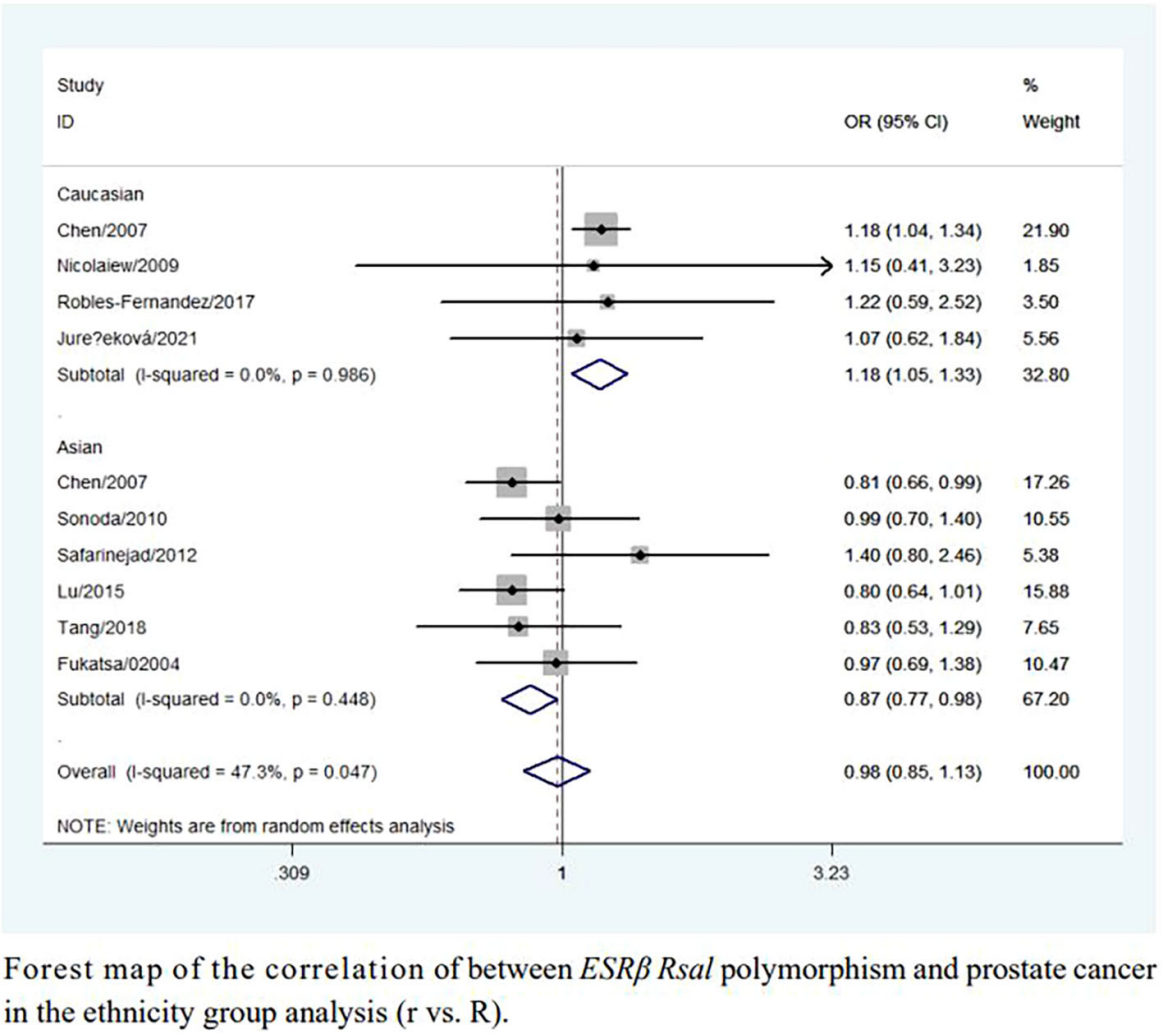
Forest map of the correlation of between *ESRβ Rsal* polymorphism and prostate cancer in the ethnicity group analysis (r vs. R).

An increased risk of prostate cancer was found in Caucasians (rr + Rr *vs.* RR: OR = 1.17, 95% CI = 1.03–1.32; r *vs.* R: OR = 1.18, 95% CI = 1.05–1.33; **Table 2**, **Figure 3**). In contrast, a reduced risk was observed in Asians (r *vs.* R: OR = 0.87, 95% CI = 0.77–0.98; **Table 2**, **Figure 3**). However, sensitivity analyses did not confirm these associations (**Table 2**).

**Table 2:** Meta-analysis of the association of *ESRβ Rsal* polymorphism with risk of prostate cancer risk.

Variable	n (Cases/Controls)	rr + Rr *vs.* RR		rr *vs.* Rr + RR		rr *vs.* RR		rr + RR *vs*. Rr		r *vs.* R	
		OR (95% CI)	*P_h_/I^2^* (%)	OR (95% CI)	*P_h_/I^2^* (%)	OR (95% CI)	*P_h_/I^2^* (%)	OR (95% CI)	*P_h_/I^2^* (%)	OR (95% CI)	*P_h_/I^2^* (%)
Overall	11 (9390/10057)	1.01(0.92-1.10)	0.079/40.5	1.14(0.87-1.51)	0.071/48.4	1.25(0.79-1.99)	0.055/51.4	1.01(0.92-1.11)	0.034/48.9	1.02(0.94-1.10)	0.071/41.7
Ethnicity											
Caucasian	4 (6708/7011)	**1.17(1.03-1.32)**	0/0	2.01(0.91-4.41)	0/0	2.03(0.92-4.45)	0/0	0.87(0.77-1.01)	0/0	**1.18(1.05-1.33)**	0/0
Asian	10 (1904/2080)	0.93(0.79-1.10)	0/0	1.17(0.66-2.06)	0.209/64.9	1.06(0.60-1.87)	0.208/63.7	1.26(0.98-1.47)	0.011/21.1	**0.87(0.77-0.98)**	0/0
Source of controls											
HB	8 (2208/2049)	0.90(0.76-1.06)	0/0	1.34(0.58-3.08)	0.424/66.3	1.26(0.55-2.89)	0.417/65.2	1.11(0.93-1.32)	0/0	0.93(0.81-1.07)	0/0
PB	3 (7182/8008)	0.96(0.70-1.30)	0.061/83.9	1.30(0.87-1.96)	0/0	1.31(0.72-2.36)	0.110/38.9	1.07(0.78-1.46)	0.064/84.1	1.01(0.79-1.28)	0.036/79.7
Sensitivity analysis											
HWE and Quality score > 8											
Overall	7 (2112/1953)	0.89 (0.75-1.06)	0.944/0	1.01(0.68-1.49)	0.031/66.3	1.26(0.55-2.89)	0.035/65.2	1.13(0.95-1.35)	0.546/0	0.93(0.80-1.07)	0.565/0
Ethnicity											
Caucasian	2 (666/339)	1.08(0.69-1.70)	0/0	2.55(0.13-49.50)	0.424/0	2.53(0.13-49.26)	0.417/0	0.96(0.61-1.52)	0/0	1.12(0.72-1.74)	0/0
Asian	5 (411/615)	0.86(0.72-1.04)	0/0	1.29(0.52-3.21)	0.490/76.5	1.21(0.49-3.00)	0.482/0	1.16(0.96-1.41)	0.02/3.9	0.90(0.78-1.05)	0/0
Source of controls											
HB	7 (2112/1953)	0.89 (0.75-1.06)	0.944/0	1.01(0.68-1.49)	0.031/66.3	1.26(0.55-2.89)	0.035/65.2	1.13(0.95-1.35)	0.546/0	0.93(0.80-1.07)	0.565/0
Egger’s test											
*P_E_*		0.865		0.586		0.961		0.694		0.72	

*ESRβ Rsal:* allele model: r vs. R, additive model: rr vs. RR, dominant model: rr + Rr vs. RR, recessive model: rr vs. RR + Rr over-dominant model: rr + RR vs. Rr; HB: hospital-based; PB: population-based. Bold values highlight the data with significant correlations.

#### ESRα Xbal and ESRβ Alul polymorphism

The overall and subgroup analyses showed no association between *ESRα* XbaI or *ESRβ* AluI polymorphisms and prostate cancer risk. Sensitivity analyses showed no significant changes in these results (**Tables 3**, **4**).

**Table 3:** Meta-analysis of the association of *ESRα Xbal* polymorphism with risk of prostate cancer risk.

Variable	n (Cases/Controls)	xx +Xx *vs.* XX		xx *vs.* Xx + XX		xx *vs.* XX		xx + XX *vs*. Xx		x *vs.* X	
		OR (95% CI)	*P_h_/I^2^* (%)	OR (95% CI)	*P_h_/I^2^* (%)	OR (95% CI)	*P_h_/I^2^* (%)	OR (95% CI)	*P_h_/I^2^* (%)	OR (95% CI)	*P_h_/I^2^* (%)
Overall	14 (3716/4536)	0.97(0.85-1.11)	0.084/36.5	0.91(0.68-1.24)	0.001/88.3	0.89(0.61-1.28)	0.001/79.2	1.00(0.80-1.25)	0.001/78.4	0.94(0.77-1.14)	0.001/85.9
Ethnicity											
Caucasian	10 (3259/3801)	0.98(0.85-1.12)	0.055/49.7	0.94(0.63-1.38)	0.328/91.5	0.90(0.58-1.41)	0.375/84.6	1.04(0.78-1.39)	0.157/84.2	0.95(0.74-1.22)	0.128/89.5
Asian	3 (375/526)	1.06(0.60-1.87)	0/0	0.90(0.68-1.19)	0/0	1.00(0.56-1.78)	0/0	1.01(0.80-1.28)	0/0	0.95(0.74-1.21)	0.007/14.5
Source of controls											
HB	11 (3348/4002)	0.95(0.79-1.14)	0.021/25.2	0.84(0.62-1.14)	0.205/86.7	0.84(0.56-1.19)	0.217/74.4	0.96(0.75-1.21)	0.108/77.6	0.90(0.74-1.11)	0.090/84.7
PB	3 (368/534)	0.93(0.38-2.32)	0.468/71.9	1.40(0.39-5.06)	1.213/94.4	1.23(0.21-7.35)	2.268/91.3	1.21(0.56-2.62)	0.403/86.5	1.10(0.51-2.37)	0.430/92.6
Sensitivity analysis											
HWE and Quality score > 8											
Overall	5 (1680/1213)	0.88(0.57-1.34)	0.015/67.6	0.98(0.55-1.77)	0.000/91.3	0.82(0.41-1.65)	0.001/85.9	1.17(0.75-1.82)	0.001/83.6	0.97(0.64-1.46)	0.001/73.6
Ethnicity											
Caucasian	4 (1563/971)	0.86(0.53-1.41)	0.176/75.5	1.02(0.50-2.10)	0.500/93.1	0.81(0.36-1.82)	0.574/89.2	1.26(0.74-2.15)	0.253/86.7	0.99(0.60-1.62)	0.235/93.1
Source of controls											
HB	4 (1563/971)	0.86(0.53-1.41)	0.176/75.5	1.02(0.50-2.10)	0.500/93.1	0.81(0.36-1.82)	0.574/89.2	1.26(0.74-2.15)	0.253/86.7	0.99(0.60-1.62)	0.235/93.1
Egger’s test											
*P_E_*		0.336		0.358		0.28		0.444		0.402	

*ESRα Xbal:* allele model: x vs. X, additive model: xx vs. XX, dominant model: xx + Xx vs. XX, recessive model: xx vs. XX + Xx over-dominant model: xx + XX vs. Xx; HB: hospital-based; PB: population-based.

**Table 4:** Meta-analysis of the association of *ESRβ Aull* polymorphism with risk of prostate cancer risk.

Variable	n (Cases/Controls)	aa + Aa *vs.* AA		aa *vs.* Aa + AA		aa *vs.* AA		aa + AA *vs*. Aa		a *vs.* A	
		OR (95% CI)	*P_h_/I^2^* (%)	OR (95% CI)	*P_h_/I^2^* (%)	OR (95% CI)	*P_h_/I^2^* (%)	OR (95% CI)	*P_h_/I^2^* (%)	OR (95% CI)	*P_h_/I^2^* (%)
Overall	8 (8674/9498)	0.99(0.93-1.05)	0.097/42.1	0.99(0.78-1.30)	0.009/62.7	0.98(0.74-1.30)	0.010/62.2	1.00(0.94-1.06)	0.113/39.9	0.99(0.89-1.10)	0.020/58
Ethnicity											
Caucasian	3 (6709/7020)	0.97(0.91-1.04)	0/0	0.98(0.88-1.06)	0/0	0.96(0.86-1.06)	0/0	1.02(0.89-1.16)	0.005/22.7	0.98(0.93-1.03)	0/0
Asian	3 (973/1147)	0.94(0.64-1.38)	0.085/74.4	0.82(0.15-4.48)	1.933/85.9	0.83(0.15-4.48)	1.886/85.4	0.97(0.65-1.44)	0.095/75.8	0.90(0.60-1.35)	0.106/83.2
Source of controls											
HB	4 (1306/1145)	0.87(0.73-1.03)	0.020/38.4	0.75(0.56-1.01)	0.232/65.8	0.67(0.38-1.17)	0.177/57.6	1.04(0.75-1.43)	0.076/70.0	0.86(0.72-1.02)	0.011/36.2
PB	4 (777/4915)	1.01(0.94-1.07)	0.006/37.6	1.00(0.92-1.10)	0.059/64.1	1.21(0.85-1.73)	0.076/ 67.5	0.99(0.93-1.06)	0/0	1.07(0.95-1.22)	0.010/92
Sensitivity analysis											
HWE and Quality score > 8											
Overall	3 (1077/906)	0.90(0.67-1.21)	0.100/56.5	0.98(0.70-1.39)	0.628/0	0.97(0.67-1.40)	0.602/0	1.11(0.83-1.48)	0.121/52.7	0.92(0.72-1.17)	0.096/57.3
Source of controls											
HB	2(858/536)	0.89(0.63-1.27)	0.313/88.6	0.88(0.55-1.38)	0.513/0	0.92(0.59-1.42)	0.371/0	1.03(0.89-1.18)	0.088/65.6	0.88(0.62-1.24)	0.034/77.4
Egger’s test											
*P_E_*		0.931		0.981		0.975		0.975		0.976	

*ESRβ Aull:* allele model: a vs. A, additive model: aa vs. AA, dominant model: aa + Aa vs. AA, recessive model: aa vs. AA + Aa over-dominant model: aa + AA vs. Aa; HB: hospital-based; PB: population-based.

### Heterogeneity and sensitivity analysis

During the statistical process, we identified several possible sources of heterogeneity were examined, including ethnicity, sample size, country, source of study population, quality score, and HWE status. Meta-regression analyses were performed, but no clear source of heterogeneity was identified. Two approaches were used for sensitivity analyses: (i) sequential removal of each study, and (ii) restricting the analysis to studies with both high quality and HWE compliance. Both approaches showed that the associations between *ESRα and ESRβ* polymorphisms and prostate cancer risk remained unchanged (**Tables 1**, **2**).

### Publication bias

**Figure 4:**
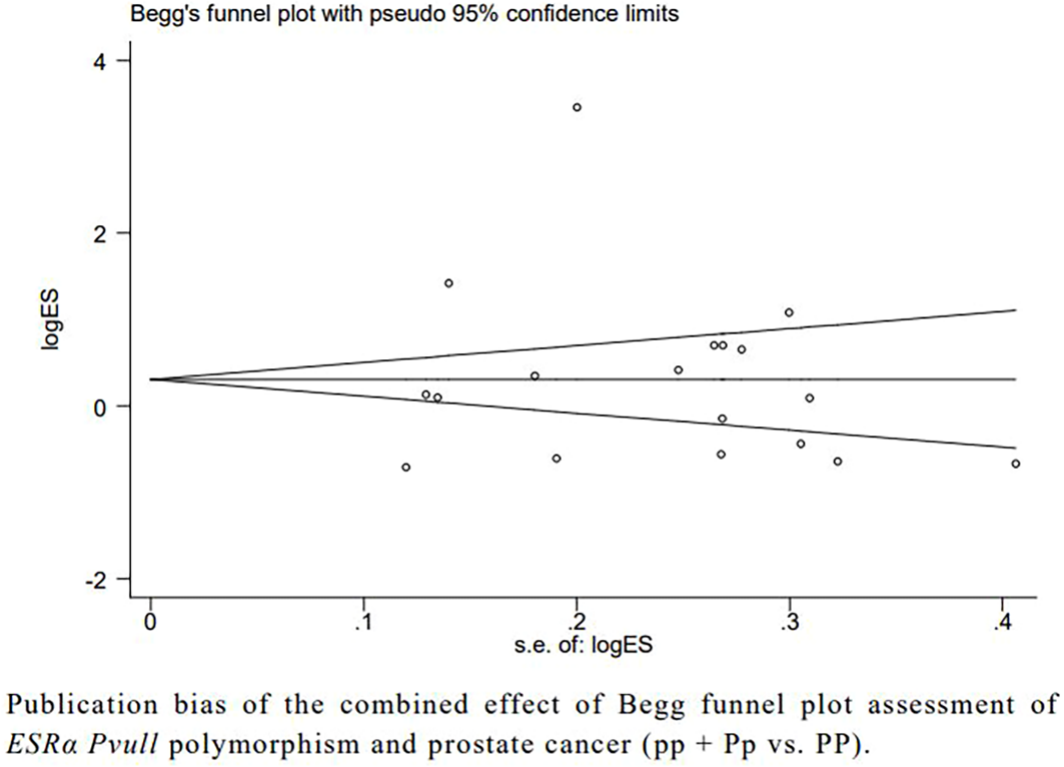
Publication bias of the combined effect of Begg funnel plot assessment of *ESRα Pvull* polymorphism anf prostate cancer (pp + Pp vs. PP).

Begg’s and Egger’s tests showed no publication bias between *ESRα Pvull*, *ESRα Xbal*, *ESRβ Alul*, and *ESRβ Rsal* polymorphisms and prostate cancer (**Figure 4**).

### Credibility analysis

The credibility of this meta-analysis was assessed using the FPRP, BFDP, and Venice criteria. In this study, we observed a reduction in the strength of all statistically significant associations (**Table 5**).

**Table 5:** Credibility of the current meta-analysis.

Gene	Variable	Model	n (Cases/Controls)	OR	P_h_/I2 (%)	Credibility		
						Prior probability of 0.001		
						Power	FPRP	BFDP
*Pvull*	Overall	pp *vs.* Pp + PP	18 (4623/9850)	0.83(0.71-0.97)	0.001/61.6	0.019	0.95	0.998
*Pvull*	Overall	pp *vs.* PP	18 (4623/9850)	0.75(0.57-0.99)	0.001/79.1	0.042	0.981	0.998
*Pvull*	Overall	p *vs.* P	18 (4623/9850)	0.88(0.78-0.99)	0.001/74.1	0.033	0.971	0.999
*Pvull*	Caucasian	pp + Pp *vs.* PP	11 (3826/8543	0.01(0.01-0.04)	0.541/20.7	0	0.981	0.246
*Pvull*	African	pp + Pp *vs.* PP	2 (129/422)	2.38(1.61-3.51)	0/0	0.001	0.551	0.378
*Pvull*	HB	pp *vs.* Pp + PP	12 (3846/4935)	0.80(0.66-0.98)	0.075/68.9	0.031	0.97	0.998
*Rsal*	Caucasian	rr + Rr *vs.* RR	4 (6708/7011)	1.17(1.03-1.32)	0/0	0.812	0.999	0.997
*Rsal*	Caucasian	r *vs.* R	4 (6708/7011)	1.18(1.05-1.33)	0/0	0.007	0.87	0.996
*Rsal*	Asian	r *vs.* R	10 (1904/2080)	0.87(0.77-0.98)	0/0	0.022	0.956	0.999
Sensitivity analysis HWE and Quality score > 8		**GG *vs.* AA**	2 (490/641)	**3.33(1.70-6.51)**	0.15/51.8	0.001	0.978	0.955
*Pvull*	Overall	pp *vs.* Pp + PP	10 (2331/6076)	0.74(0.57-0.96)	0.001/68.8	0.023	0.968	0.997
*Pvull*	Overall	pp *vs.* PP	10 (2331/6076)	0.70(0.51-0.97)	0.002/66.1	0.032	0.981	0.998
*Pvull*	Overall	p *vs.* P	10 (2331/6076)	0.86(0.74-0.98)	0.007/60.5	0.024	0.959	0.999
*Pvull*	Caucasian	pp *vs.* Pp + PP	7 (1920/5461)	0.65(0.45-0.94)	0.183/78.4	0.022	0.98	0.997
*Pvull*	Caucasian	pp *vs.* PP	7 (1920/5461)	0.61(0.39-0.95)	0.257/76.5	0.029	0.988	0.997
*Pvull*	Caucasian	p *vs.* P	7 (1920/5461)	0.81(0.66-0.98)	0.047/72.4	0.03	0.969	0.998
*Pvull*	HB	pp *vs.* Pp + PP	6 (1792/1568)	0.68(0.46-0.99)	0.027/35	0.007	0.926	0.993

HB= hospital-based; PB: population-based. Bold values highlight the data with significant correlations.

## Discussion

Interestingly, the effect of *ESR* on the risk of prostate cancer differed significantly across ethnic groups. We found that *ESRβ Rsal* was associated with a significantly increased risk of prostate cancer in Caucasians. However, *ESRβ Rsal* appears to be a protective gene against prostate cancer in Asians. Meanwhile, *Pvull* may also be a risk gene locus for prostate cancer in Africans. However, *ESRα Pvull* gene polymorphism showed a significantly reduced association with prostate cancer risk in the general population. However, no significant results were found in the final sensitivity analyses. Although we attempted to include all relevant studies, the inclusion of low-quality studies may have affected the true associations. Unfortunately, we did not find an association between *ESRα Xbal* and *ESRβ Alul* and the risk of prostate cancer.

We used subgroups of race and mode of recruitment, as well as five genetic models, which necessarily led to multiple comparisons; therefore, adjustment for pooled p-values was necessary. The confidence of the associations between *ESRα Pvull*, *Xbal*, and *ESRβ Alul*, *Rsal* polymorphisms and prostate cancer risk was low according to the FPRP, BFDP, and Venice criteria. This seemingly contradictory outcome primarily stems from the following aspects. First, the calculation of FPRP and BFDP depends not only on the P-value but, more critically, on statistical power and the pre-specified prior probability. In this study, the statistical power for this specific variant was at a moderate level, which increases the relative risk of the result being a false positive. Second, adhering to a principle of caution, we employed a relatively conservative prior probability (0.001) in our calculations. This reflects a more prudent stance toward this finding, given the current lack of strong biological or genetic evidence. Therefore, the “low confidence” rating does not negate the association but emphasizes that our findings should be considered preliminary and suggestive evidence, whose ultimate confirmation will require replication in larger, independent cohorts or prospective studies. Future research should aim to validate the association observed here in samples with sufficient statistical power, complemented by functional experimental evidence. 

Based on subgroup analyses by population, the results across different ethnic subgroups revealed contradictory patterns. Our findings underscore that the effects of genetic variants may be highly population-specific. The underlying factors for these discrepancies may include differences in allele frequencies, variations in linkage disequilibrium patterns across populations, and gene–environment interactions. Extreme caution should be exercised when extrapolating these findings to populations with different genetic backgrounds. Future studies should focus on more refined genetic analyses within individual populations and integrate environmental exposure data to elucidate the precise mechanisms underlying these discordant associations. 

Previously, eight interlinked meta-analyses had published results on the association between the *ESRα Pvull*, *Xbal*, and *ESRβ Alul*, *Rsal* genetic polymorphisms and the risk of prostate cancer. Chang et al, Fu et al, Ma et al, and Liu found that *ESRβ Rsal* might be correlated with an increased risk of PCa in Caucasians, while less susceptibility was observed in Asians (8, 9, 13, 14). Wang et al. and Li et al. found that the *PvuII* polymorphism was significantly associated with an increased risk of prostate cancer among Asian populations (11, 15). Gazzaz et al. demonstrated that *ESRα Rsal* increases the risk of prostate cancer. Gu et al. suggested that *ESRα Xbal* is a noteworthy gene for prostate cancer risk in Africans (12). Unfortunately, we did not replicate the findings of Gu et al. after including additional data from our study. The conflicting results, particularly in Asian populations, may be due to the insufficient number of included studies. These studies examined only the association between a single SNP and prostate cancer risk. Furthermore, several studies did not conduct a quality assessment of eligible articles. In addition, none evaluated the credibility of statistically significant associations. Compared with previous studies, our study is the first to reveal a significant association between the ESRα Pvull polymorphism and a reduced risk of prostate cancer. By including a larger number of studies, we further clarified the relationship between ESRα, ESRβ, and prostate cancer risk.

In order to further clarify the uncertain results of previous meta-analyses and evaluate current findings, we conducted this meta-analysis. The present study has the following advantages over previous analyses: (i) strict inclusion criteria; (ii) HWE examination of control groups; (iii) assessment of the credibility of statistically significant associations; (iv) inclusion of more studies with larger sample sizes; and (v) more accurate subgroup analyses and more professional modeling approaches. The shortcomings of previous studies were noted and addressed. However, our study has several limitations. First, we included only published articles that could be retrieved through database searches. Second, the included studies did not adjust for confounding factors that may significantly affect the results; therefore, our findings are based on unadjusted OR estimates. Third, although the overall sample size was large, the quality of the included studies varied, which may reduce confidence in the final results. Therefore, additional studies are needed in the future to further verify our findings.

The pathogenesis of prostate cancer involves complex interactions between environmental factors and polygenic influences. While our investigation specifically examined the impact of *ESR* single-nucleotide polymorphisms on prostate cancer risk, this systematic analysis incorporated the majority of currently available studies on the ESR**–**prostate cancer association. Our findings further elucidate the relationship between ESR and prostate cancer and provide a meaningful perspective for future prostate cancer research and the identification of potential therapeutic targets.

## Conclusion

In summary, *ESRα Pvull* and *ESRβ Rsal* polymorphisms are associated with the risk of prostate cancer. However, the observed effects were inconsistent or weak across studies. To resolve this uncertainty and confirm any potential link, large studies with sufficient methodological quality are needed in the future.

## Data Availability

The original contributions presented in the study are included in the article/**Supplementary Material**. Further inquiries can be directed to the corresponding authors.

## References

[1] Bergengren O Pekala KR Matsoukas K Fainberg J Mungovan SF Bratt O . 2022 update on prostate cancer epidemiology and risk factors-A systematic review. Eur Urol. (2023) 84:191–206. doi: 10.1016/j.eururo.2023.04.021, PMID: 37202314 PMC10851915

[2] Ju W Zheng R Zhang S Zeng H Sun K Wang S . Cancer statistics in Chinese older people, 2022: current burden, time trends, and comparisons with the US, Japan, and the Republic of Korea. Sci China Life Sci. (2023) 66:1079–91. doi: 10.1007/s11427-022-2218-x, PMID: 36543994

[3] Baca SC Singler C Zacharia S Seo JH Morova T Hach F . Genetic determinants of chromatin reveal prostate cancer risk mediated by context-dependent gene regulation. Nat Genet. (2022) 54:1364–75. doi: 10.1038/s41588-022-01168-y, PMID: 36071171 PMC9784646

[4] Hassan NE El Shebini SM El-Masry SA Ahmed NH Eldeen GN Rasheed EA . Association of some dietary ingredients, vitamin D, estrogen, and obesity polymorphic receptor genes with bone mineral density in a sample of obese Egyptian women. J Genet Eng Biotechnol. (2021) 19:28. doi: 10.1186/s43141-021-00127-0, PMID: 33559788 PMC7873164

[5] Lambert MNT Hu LM Jeppesen PB . A systematic review and meta-analysis of the effects of isoflavone formulations against estrogen-deficient bone resorption in peri- and postmenopausal women. Am J Clin Nutr. (2017) 106:801–11. doi: 10.3945/ajcn.116.151464, PMID: 28768649

[6] Kos M Reid G Denger S Gannon F . Minireview: genomic organization of the human ERalpha gene promoter region. Mol Endocrinol. (2001) 15:2057–63. doi: 10.1210/me.15.12.2057, PMID: 11731608

[7] Maguire P Margolin S Skoglund J Sun XF Gustafsson JA Borresen-Dale AL . Estrogen receptor beta (ESR2) polymorphisms in familial and sporadic breast cancer. Breast Cancer Res Treat. (2005) 94:145–52. doi: 10.1007/s10549-005-7697-7, PMID: 16261413

[8] Chang X Wang H Yang Z Wang Y Li J Han Z . ESR2 polymorphisms on prostate cancer risk: A systematic review and meta-analysis. Med (Baltimore). (2023) 102:e33937. doi: 10.1097/MD.0000000000033937, PMID: 37335680 PMC10256358

[9] Fu C Dong WQ Wang A Qiu G . The influence of ESR1 rs9340799 and ESR2 rs1256049 polymorphisms on prostate cancer risk. Tumour Biol. (2014) 35:8319–28. doi: 10.1007/s13277-014-2086-7, PMID: 24859835

[10] Gazzaz H El Feniche M Ameur A Dami A . Association between the Estrogen receptor β rs1256049 polymorphism and prostate cancer risk:a meta-analysis. Ann Biol Clin (Paris). (2023) 81:280–8. doi: 10.1684/abc.2023.1815, PMID: 37329151

[11] Wang YM Liu ZW Guo JB Wang XF Zhao XX Zheng X . ESR1 gene polymorphisms and prostate cancer risk: A huGE review and meta-analysis. PloS One. (2013) 8:e66999. doi: 10.1371/journal.pone.0066999, PMID: 23805288 PMC3689664

[12] Gu Z Wang G Chen W . Estrogen receptor alpha gene polymorphisms and risk of prostate cancer: a meta-analysis involving 18 studies. Tumour Biol. (2014) 35:5921–30. doi: 10.1007/s13277-014-1785-4, PMID: 24584714

[13] Liu P . A meta-analysis of estrogen receptor β gene rs1256049 polymorphism and prostate cancer susceptibility. No. 382, Wuyi Road, Xinghualing District, Taiyuan City, Shanxi Province: Shanxi Medical University (2019).

[14] Ma Y-d Wang A-N Zhong S-p . Relationship between estrogen receptor α gene rs2234693 polymorphism and risk of prostate cancer. Chin J Physiol. (2015) 1):104–8.

[15] Li L Zhang X Xia Q Ma H Chen L Hou W . Association between estrogen receptor alpha PvuII polymorphism and prostate cancer risk. Tumour Biol. (2014) 35:4629–35. doi: 10.1007/s13277-014-1606-9, PMID: 24414486

[16] Wang D Liu L Zhang C Lu W Wu F He X . Evaluation of association studies and meta-analyses of *eNOS* polymorphisms in type 2 diabetes mellitus risk. Front Genet. (2022) 13:887415. doi: 10.3389/fgene.2022.887415, PMID: 35832187 PMC9271911

[17] Mu YY Liu B Chen B Zhu WF Ye XH Li HZ . Evaluation of association studies and an updated meta-analysis of VDR polymorphisms in osteoporotic fracture risk. Front Genet. (2022) 12:791368. doi: 10.3389/fgene.2021.791368, PMID: 35069689 PMC8782145

[18] Modugno F Weissfeld JL Trump DL Zmuda JM Shea P Cauley JA . Allelic variants of aromatase and the androgen and estrogen receptors:toward a multigenic model of prostate cancer risk. Clin Cancer Res. (2001) 7:3092–6., PMID: 11595700

[19] Tanaka Y Sasaki M Kaneuchi M Shiina H Igawa M Dahiya R . Polymorphisms estrogen receptor alpha in prostate cancer. Mol Carcinog. (2003) 37:202–8. doi: 10.1002/mc.10138, PMID: 12891629

[20] Fukatsu T Hirokawa Y Araki T Hioki T Murata T Suzuki H . Genetic polymorphisms of hormone-related genes and prostate cancer risk in the Japanese population. Anticancer Res. (2004) 24:2431–7., PMID: 15330195

[21] Hernandez J Balic I Johnson-Pais TL Higgins BA Torkko KC Thompson IM . Association between an estrogen receptor alpha gene polymorphism and the risk of prostate cancer in black men. J Urol. (2006) 175:523–7. doi: 10.1016/S0022-5347(05)00240-5, PMID: 16406987

[22] Low YL Taylor JI Grace PB Mulligan AA Welch AA Scollen S . Phytoestrogen exposure, polymorphisms in COMT, CYP19, ESR1, and SHBG genes, and their associations with prostate cancer risk. Nutr Cancer. (2006) 56:31–9. doi: 10.1207/s15327914nc5601_5, PMID: 17176215

[23] Kjaergaard AD Ellervik C Tybjaerg-Hansen A Axelsson CK Grønholdt ML Grande P . Estrogen receptor alpha polymorphism and risk of cardio -vascular disease, cancer, and hip fracture: cross-sectional, cohort, and case-control studies and a meta-analysis. Circulation. (2007) 115:861–71. doi: 10.1161/CIRCULATIONAHA.106.615567, PMID: 17309937

[24] Cunningham JM Hebbring SJ McDonnell SK Cicek MS Christensen GB Wang L . Evaluation of genetic variations in the androgen and estro -gen metabolic pathways as risk factors for sporadic and fa -milial prostate cancer. Cancer Epidemiol Biomarkers Prev. (2007) 16:969–78. doi: 10.1158/1055-9965.EPI-06-0767, PMID: 17507624

[25] Berndt SI Chatterjee N Huang WY Chanock SJ Welch R Crawford ED . Variant in sex hormone-binding globulin gene and the risk of prostate. Cancer Epidemiol Biomarkers Prev. (2007) 16:165–8. doi: 10.1158/1055-9965.EPI-06-0689, PMID: 17220347

[26] Onsory K Sobti RC Al-Badran AI Watanabe M Shiraishi T Krishan A . Hormone receptor-related gene polymorphisms and prostate cancer risk in the Northern Indian population. Mol Cell Biochem. (2008) 314:25–35. doi: 10.1007/s11010-008-9761-1, PMID: 18483761

[27] Beuten J Gelfond JA Franke JL Weldon KS Crandall AC Johnson-Pais TL . Single and multigenic analysis of the association between variants in 12 steroid hormone metabolism genes and risk of prostate. Cancer Epidemiol Biomarkers Prev. (2009) 18:1869–80. doi: 10.1158/1055-9965.EPI-09-0076, PMID: 19505920

[28] Gupta L Thakur H Sobti RC Seth A Singh SK . Role of genetic po syndrome lymorphism of estrogen receptor-alpha gene and risk of estrogen receptor alpha prostate cancer in north Indian population. Mol Cell Biochem. (2010) 335:255–61. doi: 10.1007/s11010-009-0275-2, PMID: 19904497

[29] Sonoda T Suzuki H Mori M Tsukamoto T Yokomizo A Naito S . Polymorphisms in estrogen related genes may modify the protective effect isoflavones against prostate cancer risk in Japanese men. Eur J Cancer Prev. (2010) 19:131–7. doi: 10.1097/CEJ.0b013e328333fbe2, PMID: 19952760

[30] Sissung TM Danesi R Kirkland CT Baum CE Ockers SB Stein EV . Estrogen receptor αand aromatase polymorphisms affect risk, prognosis, and therapeutic outcome in men with castration-resist ant prostate cancer treated with docetaxel-based therapy. J Clin Endocrinol Metab. (2011) 96:E368–72. doi: 10.1210/jc.2010-2070, PMID: 21106711 PMC3048329

[31] Szendroi A Speer G Tabak A Kosa JP Nyirady P Majoros A . The role of vitamin D, estrogen, calcium sensing receptor genotypes andserum calcium in the pathogenesis of prostate cancer. Can J Urol. (2011) 18:5710–6., PMID: 21703046

[32] Safarinejad MR Safarinejad S Shafiei N Safarinejad S . Estrogen receptors alpha (rs2234693 and rs9340799), and beta (rs4986938 and rs1256049) gene polymorphism in prostate cancer: evidence for association with risk and his -Pathological characteristics of tumor in Iranian men. Mol Carcinog. (2012) 51:E104–17. doi: 10.1002/mc.21870, PMID: 22228197

[33] Jurečeková J Sivoňová MK Evinová A Kliment J Dobrota D . The association between estrogen receptor alpha polymorphisms and the risk of prostate cancer in Slovak population. Mol Cell Biochem. (2013) 381:201–7. doi: 10.1007/s11010-013-1703-x, PMID: 23737135

[34] Suzuki K Nakazato H Matsui H Koike H Okugi H Kashiwagi B . Genetic polymorphisms of estrogen receptor alpha, CYP19, catechol-O-methyltransferase are associated with familial prostate carcinoma risk in a Japanese population. Cancer. (2003) 98:411–6. doi: 10.1002/cncr.11639, PMID: 14508827

[35] Balistreri CR Caruso C Carruba G Miceli V Candore G . Genotyping of sex hormone-related pathways in benign and Malignant human prostate tissues: data of a preliminary study. OMICS. (2011) 5:369–74. doi: 10.1089/omi.2010.0128, PMID: 21348640

[36] Chae YK Huang HY Strickland P Hoffman SC Helzlsouer K . Genetic polymorphisms of estrogen receptors alpha and beta and the risk of developing prostate cancer. PloS One. (2009) 4:6523. doi: 10.1371/journal.pone.0006523, PMID: 19654868 PMC2715882

[37] Lu X Yamano Y Takahashi H Koda M Fujiwara Y Hisada A . Associations between estrogen receptor genetic polymorphisms, smoking status, and prostate cancer risk: a case-control study in Japanese men. Environ Health Prevent Med. (2015) 20:32–7. doi: 10.1007/s12199-015-0471-5, PMID: 26251204 PMC4550607

[38] Chen YC Kraft P Bretsky P Ketkar S Hunter DJ Albanes D . Sequence variants of estrogen receptor beta and risk of prostate cancer in the National Cancer Institute Breast and Prostate Cancer Cohort Consortium. Cancer Epidemiol Biomarkers Prevent. (2007) 6:1973–81. doi: 10.1158/1055-9965.EPI-07-0431, PMID: 17932344

[39] Nicolaiew N Cancel-Tassin G Azzouzi AR Grand BL Mangin P Cormier L . Association between estrogen and androgen receptor genes and prostate cancer risk. Eur J Endocrinol. (2009) 160:01–6. doi: 10.1530/EJE-08-0321, PMID: 18952763

[40] Robles-Fernandez I Martinez-Gonzalez LJ Pascual-Geler M Cozar JM Puche-Sanz I Serrano MJ . Association between polymorphisms in sex hormones synthesis and metabolism and prostate cancer aggressiveness. PloS One. (2017) 12:01 85447e0185447. doi: 10.1371/journal.pone.0185447, PMID: 28981526 PMC5628818

[41] Tang L Platek ME Yao S Till C Goodman PJ Tangen CM . Associations between polymorphisms in genes related to estrogen metabolism and function and prostate cancer risk: results from the prostate cancer prevention trial. Carcinogenesis. (2018) 9:125–33. doi: 10.1093/carcin/bgx144, PMID: 29228205 PMC6075364

[42] Moher D Liberati A Tetzlaff J Altman DG PRISMA Group . Preferred reporting items for systematic reviews and meta-analyses: the PRISMA statement. PloS Med. (2009) 6:e1000097. doi: 10.1371/journal.pmed.1000097, PMID: 19621072 PMC2707599

[43] Thakkinstian A McKay GJ McEvoy M Chakravarthy U Chakrabarti S Silvestri G . Systematic review and meta-analysis of the association between complement component 3 and age-related macular degeneration: a HuGE review and meta-analysis. Am J Epidemiol. (2011) 173:1365–79. doi: 10.1093/aje/kwr025, PMID: 21576320

[44] Mantel N Haenszel W . Statistical aspects of the analysis of data from retrospective studies of disease. J Natl Cancer Inst. (1959) 22:719–48. 13655060

[45] DerSimonian R Laird N . Meta-analysis in clinical trials revisited. Contemp Clin Trials. (2015) 45:139–45. doi: 10.1016/j.cct.2015.09.002, PMID: 26343745 PMC4639420

[46] Egger M Davey Smith G Schneider M Minder C . Bias in meta-analysis detected by a simple, graphical test. BMJ. (1997) 315:629–34. doi: 10.1136/bmj.315.7109.629, PMID: 9310563 PMC2127453

[47] Begg CB Mazumdar M . Operating characteristics of a rank correlation test for publication bias. Biometrics. (1994) 50:1088–101. doi: 10.2307/2533446, PMID: 7786990

[48] Wacholder S Chanock S Garcia-Closas M El Ghormli L Rothman N . Assessing the probability that a positive report is false: an approach for molecular epidemiology studies. J Natl Cancer Inst. (2004) 96:434–42. doi: 10.1093/jnci/djh075, PMID: 15026468 PMC7713993

[49] Wakefield J . A Bayesian measure of the probability of false discovery in genetic epidemiology studies. Am J Hum Genet. (2007) 81:208–27. doi: 10.1086/519024, PMID: 17668372 PMC1950810

[50] Ioannidis JP Boffetta P Little J O'Brien TR Uitterlinden AG Vineis P . Assessment of cumulative evidence on genetic associations: interim guidelines. Int J Epidemiol. (2008) 37:120–32. doi: 10.1093/ije/dym159, PMID: 17898028

