# Supplementary material for: Association between ESRα and ESRβ polymorphisms and prostate cancer risk: meta-analysis
Source: Front Oncol. 2025 Dec 8;15:1630363. doi: 10.3389/fonc.2025.1630363 (PMC12719423; doi:10.3389/fonc.2025.1630363)
Supplement: Supplementary file 1 [file Table1.docx]

Supplementary Table 1. Scale for quality assessment of molecular association studies

| Source of case | |
| --- | --- |
| Selected from population | 2 |
| Selected from hospital | 1 |
| Not described | 0 |
| Source of control | |
| Population-based | 2 |
| Hospital-based | 1 |
| Not described | 0 |
| Matching | |
| Controls matched with cases by age and sex | 2 |
| Controls matched with cases only by age or sex | 1 |
| Not matched or not described | 0 |
| Genotyping examination | |
| Genotyping done blindly and quality control | 2 |
| Only genotyping done blindly or quality control | 1 |
| Unblinded and without quality control | 0 |
| Specimens used for determining genotypes | |
| Blood cells or normal tissues | 1 |
| Not described | 0 |
| HWE ((Hardy-Weinberg Equilibrium) | |
| HWE in the control group | 2 |
| Hardy-Weinberg disequilibrium in the control group | 0 |
| Association assessment | |
| Assess association between genotypes and male infertility with appropriate statistics and adjustment for confounders | 2 |
| Assess association between genotypes and male infertility with appropriate statistics without adjustment for confounders | 1 |
| Inappropriate statistics used | 0 |
| Total sample size | |
| >500 | 3 |
| 200-500 | 2 |
| <200 | 1 |
