# Supplementary material for: Association between ESRα and ESRβ polymorphisms and prostate cancer risk: meta-analysis
Source: Front Oncol. 2025 Dec 8;15:1630363. doi: 10.3389/fonc.2025.1630363 (PMC12719423; doi:10.3389/fonc.2025.1630363)
Supplement: Supplementary file 2 [file Table2.docx]

| **S2 Table General characteristic and the results of the included meta-analyses on the *ESRα Pvull* gene polymorphism with prostate risk** | | | | | | | | | | | | | | |
| --- | --- | --- | --- | --- | --- | --- | --- | --- | --- | --- | --- | --- | --- | --- |
| **First author/Year** | **Country** | **Ethnicity** | **Type of controls** |  | | | | | | | | | | |
|  |  |  |  | **HWE** | | **Number of samples** | | | **Genotypes of cases** | | | **Genotypes of controls** | | |
|  |  |  |  | **P** | | **Cases** | **Controls** | **Total** | **PP** | **Pp** | **pp** | **PP** | **Pp** | **pp** |
| Modugno/2001 | USA | Caucasian | PB | 0.438 |  | 81 | 237 | 318 | 21 | 34 | 36 | 43 | 109 | 85 |
| Tanaka/2003 | USA | Asian | PB | 0.061 |  | 115 | 200 | 315 | 29 | 63 | 23 | 48 | 113 | 39 |
| Fukatsa/02004 | Japan | Asian | HB | 0.384 |  | 116 | 238 | 354 | 22 | 57 | 37 | 47 | 110 | 81 |
| Hernandez/2006 | USA | Caucasian | HB | 0.455 |  | 551 | 885 | 1436 | 118 | 271 | 162 | 145 | 427 | 283 |
| Hernandez/2006 | USA | African | HB | 0.373 |  | 47 | 213 | 260 | 16 | 22 | 9 | 50 | 113 | 50 |
| Low/2006 | UK | Caucasian | HB | 0.266 |  | 75 | 158 | 233 | 21 | 41 | 13 | 25 | 84 | 49 |
| Kjaergaard/2007 | Danish | Caucasian | PB | 0.676 |  | 116 | 4005 | 4121 | 26 | 55 | 35 | 830 | 1972 | 1203 |
| Cunningham/2007 | China | Caucasian | HB | 0.684 |  | 924 | 489 | 1413 | 213 | 454 | 257 | 120 | 249 | 120 |
| Berndt/2007 | USA | Caucasian | HB | 0.230 |  | 470 | 603 | 1073 | 111 | 238 | 121 | 135 | 316 | 152 |
| Onsory/2008 | India | Asian | HB | 0.487 |  | 100 | 100 | 200 | 18 | 54 | 28 | 10 | 48 | 42 |
| Beuten/2009 | USA | African | HB | 0.941 |  | 82 | 209 | 291 | 23 | 41 | 18 | 50 | 105 | 54 |
| Beuten/2009 | USA | Caucasian | HB | 0.957 |  | 804 | 1357 | 2161 | 66 | 396 | 242 | 282 | 667 | 408 |
| Gupta/2010 | India | Asian | PB | 0.0496 |  | 157 | 170 | 327 | 28 | 77 | 52 | 16 | 90 | 64 |
| Sonada/2010 | Japan | Asian | PB | 0.829 |  | 180 | 177 | 357 | 31 | 89 | 60 | 29 | 87 | 61 |
| Sissung/2010 | USA | Caucasian | PB | 0.953 |  | 128 | 126 | 254 | 28 | 75 | 25 | 20 | 60 | 46 |
| Szendroi/2011 | Hungary | Caucasian | HB | 0.392 |  | 204 | 103 | 307 | 39 | 122 | 43 | 25 | 47 | 31 |
| Safarinejad/2012 | Iranian | Caucasian | HB | 0.373 |  | 162 | 324 | 486 | 57 | 94 | 11 | 90 | 169 | 65 |
| Jurecekova//2013 | Slovak | Caucasian | HB | 1.000 |  | 311 | 256 | 567 | 79 | 154 | 78 | 49 | 126 | 81 |
|  |  |  |  |  |  |  |  |  |  |  |  |  |  |  |
