# Supplementary material for: Association between ESRα and ESRβ polymorphisms and prostate cancer risk: meta-analysis
Source: Front Oncol. 2025 Dec 8;15:1630363. doi: 10.3389/fonc.2025.1630363 (PMC12719423; doi:10.3389/fonc.2025.1630363)
Supplement: Supplementary file 3 [file Table3.docx]

| **S3 Table General characteristic and the results of the included meta-analyses on the *ESRα Xbal* gene polymorphism with prostate risk** | | | | | | | | | | | | | |
| --- | --- | --- | --- | --- | --- | --- | --- | --- | --- | --- | --- | --- | --- |
| First author/Year | Country | Ethnicity | Type of controls |  | | | | | | | | | |
|  |  |  |  | HWE | Number of samples | | | Genotypes of cases | | | Genotypes of controls | | |
|  |  |  |  | P | Cases | Controls | Total | XX | Xx | xx | XX | Xx | xx |
| Modugno/2001 | USA | Caucasian | PB | 1.64 | 82 | 237 | 319 | 10 | 38 | 41 | 28 | 93 | 16 |
| Suzuki/2003 | Japan | Asian | HB | 0.026 | 101 | 114 | 215 | 5 | 24 | 72 | 9 | 30 | 75 |
| Fukatsa/02004 | Japan | Asian | HB | 0.264 | 117 | 242 | 359 | 6 | 37 | 74 | 11 | 68 | 163 |
| Hernandez/2006 | USA | Caucasian | HB | 0.513 | 551 | 795 | 1346 | 71 | 242 | 248 | 103 | 400 | 352 |
| Hernandez/2006 | USA | Caucasian | HB | 0.223 | 47 | 213 | 260 | 5 | 25 | 17 | 19 | 77 | 117 |
| Cunningham/2007 | China | Caucasian | HB | 0.832 | 918 | 487 | 1405 | 121 | 417 | 380 | 71 | 227 | 189 |
| Beuten/2009 | USA | African | HB | 0.982 | 82 | 209 | 291 | 9 | 36 | 37 | 13 | 78 | 118 |
| Beuten/2009 | USA | Caucasian | HB | 0.142 | 804 | 1214 | 2018 | 94 | 361 | 349 | 126 | 481 | 559 |
| Gupta/2010 | India | Asian | PB | 0.444 | 157 | 170 | 327 | 11 | 75 | 71 | 11 | 72 | 87 |
| Sissung/2010 | USA | Caucasian | PB | 0.123 | 129 | 127 | 256 | 18 | 69 | 42 | 8 | 61 | 58 |
| Balistreri 2011 | Spain | Caucasian | HB | 0.048 | 50 | 47 | 97 | 3 | 13 | 34 | 1 | 4 | 42 |
| Szendroi/2011 | Hungary | Caucasian | HB | 0.409 | 205 | 101 | 306 | 59 | 111 | 35 | 18 | 54 | 29 |
| Safarinejad/2012 | Iranian | Caucasian | HB | 0.004 | 162 | 324 | 486 | 34 | 108 | 20 | 56 | 187 | 81 |
| Jurecekova//2013 | Slovak | Caucasian | HB | 5.08 | 311 | 256 | 567 | 56 | 32 | 145 | 105 | 110 | 119 |
|  |  |  |  |  |  |  |  |  |  |  |  |  |  |
