# Supplementary material for: Association between ESRα and ESRβ polymorphisms and prostate cancer risk: meta-analysis
Source: Front Oncol. 2025 Dec 8;15:1630363. doi: 10.3389/fonc.2025.1630363 (PMC12719423; doi:10.3389/fonc.2025.1630363)
Supplement: Supplementary file 4 [file Table4.docx]

| ***S*4 Table General characteristic and the results of the included meta-analyses on the *ESRβ Aull* gene polymorphism with prostate cancer risk** | | | | | | | | | | | | | |
| --- | --- | --- | --- | --- | --- | --- | --- | --- | --- | --- | --- | --- | --- |
| First author/Year | Country | Ethnicity | Type of controls |  | | | | | | | | | |
|  |  |  |  | HWE | Number of samples | | | Genotypes of cases | | | Genotypes of controls | | |
|  |  |  |  | P | Cases | Controls | Total | AA | Aa | aa | AA | Aa | aa |
| Chae | USA | Mix | PB | 0.308 | 219 | 370 | 589 | 81 | 105 | 33 | 134 | 185 | 51 |
| Chen/2007 | USA | African | PB | 0.791 | 773 | 961 | 1734 | 408 | 300 | 65 | 538 | 360 | 63 |
| Chen/2007 | USA | Caucasian | PB | 0.048 | 5917 | 6551 | 12468 | 2274 | 2739 | 904 | 2481 | 3039 | 1031 |
| Chen/2007 | USA | Asian | PB | 0.025 | 459 | 471 | 930 | 315 | 131 | 13 | 346 | 122 | 3 |
| Jurecekova/2021 | Slovakia | Caucasian | HB | 0.723 | 506 | 184 | 690 | 228 | 229 | 49 | 88 | 77 | 19 |
| Lu/2015 | Japan | Asian | HB | 0.993 | 352 | 352 | 704 | 280 | 67 | 5 | 254 | 90 | 8 |
| Nicolaiew/2009 | France | Caucasian | HB | 0.034 | 286 | 285 | 571 | 138 | 100 | 48 | 122 | 116 | 47 |
| Safarinejad/2012 | Iran | Asian | HB | 0.034 | 162 | 324 | 486 | 81 | 76 | 5 | 159 | 124 | 41 |
|  |  |  |  |  |  |  |  |  |  |  |  |  |  |
