# Supplementary material for: Association between ESRα and ESRβ polymorphisms and prostate cancer risk: meta-analysis
Source: Front Oncol. 2025 Dec 8;15:1630363. doi: 10.3389/fonc.2025.1630363 (PMC12719423; doi:10.3389/fonc.2025.1630363)
Supplement: Supplementary file 5 [file Table5.docx]

| **S5 Table General characteristic and the results of the included meta-analyses on the *ESRβ Rsal* gene polymorphism with prostate cancer risk** | | | | | | | | | | | | | | |
| --- | --- | --- | --- | --- | --- | --- | --- | --- | --- | --- | --- | --- | --- | --- |
| First author/Year | Country | Ethnicity | Type of controls |  | | | | | | | | | | |
|  |  |  |  | HWE | | Number of samples | | | Genotypes of cases | | | Genotypes of controls | | |
|  |  |  |  | P | | Cases | Controls | Total | RR | Rr | rr | RR | Rr | rr |
| Chen/2007 | USA | African | PB | 0.396 |  | 778 | 966 | 1744 | 657 | 115 | 6 | 819 | 143 | 4 |
| Chen/2007 | USA | Asian | PB | 0.049 |  | 458 | 466 | 924 | 259 | 166 | 33 | 222 | 212 | 32 |
| Chen/2007 | USA | Caucasian | PB | 0.975 |  | 5946 | 6576 | 12522 | 5442 | 488 | 16 | 6096 | 471 | 9 |
| Nicolaiew/2009 | France | Caucasian | HB | 0.711 |  | 96 | 96 | 192 | 88 | 8 | 0 | 89 | 7 | 0 |
| Sonoda/2010 | Japan | Asian | HB | 4.25 |  | 180 | 176 | 356 | 96 | 84 | 0 | 93 | 83 | 0 |
| Safarinejad/2012 | Iran | Asian | HB | 1.43 |  | 162 | 324 | 486 | 150 | 2 | 10 | 300 | 16 | 8 |
| Lu/2015 | Japan | Asian | HB | 0.409 |  | 352 | 352 | 704 | 185 | 142 | 25 | 167 | 146 | 39 |
| Robles-Fernandez/2017 | Spain | Caucasian | HB | 0.556 |  | 156 | 155 | 311 | 139 | 17 | 0 | 141 | 14 | 0 |
| Tang/2018 | USA | Asian | HB | 0.352 |  | 616 | 526 | 1142 | 576 | 40 | 0 | 485 | 41 | 0 |
| Jurečeková/2021 | Slovakia | Caucasian | HB | 0.485 |  | 510 | 184 | 694 | 460 | 47 | 3 | 166 | 18 | 0 |
| Fukatsa/02004 | Japan | Asina | HB | 0.478 |  | 136 | 236 | 372 | 82 | 43 | 11 | 133 | 91 | 12 |
|  |  |  |  |  |  |  |  |  |  |  |  |  |  |  |
