# Supplementary material for: Association between ESRα and ESRβ polymorphisms and prostate cancer risk: meta-analysis
Source: Front Oncol. 2025 Dec 8;15:1630363. doi: 10.3389/fonc.2025.1630363 (PMC12719423; doi:10.3389/fonc.2025.1630363)
Supplement: Supplementary file 6 [file Table6.docx]

| **S6 Table cale for quality assessment of molecular association studies** | | | | | | | | | |
| --- | --- | --- | --- | --- | --- | --- | --- | --- | --- |
| First author/Year | Source of case | Source of control | Matching | Genotyping examination | Specimens used for determining genotypes | HWE ((Hardy-Weinberg Equilibrium) | Association assessment | Total sample size | Toal score |
| Modugno/2001 | 1 | 2 | 1 | 0 | 0 | 2 | 0 | 2 | 8 |
| Tanaka/2003 | 1 | 2 | 1 | 1 | 1 | 2 | 1 | 2 | 11 |
| Fukatsa/02004 | 1 | 1 | 1 | 1 | 1 | 2 | 1 | 2 | 10 |
| Hernandez/2006 | 1 | 1 | 0 | 1 | 0 | 2 | 0 | 3 | 8 |
| Low/2006 | 1 | 1 | 1 | 1 | 1 | 2 | 1 | 2 | 10 |
| Kjaergaard/2007 | 1 | 2 | 1 | 1 | 1 | 2 | 1 | 3 | 12 |
| Cunningham/2007 | 1 | 1 | 1 | 1 | 0 | 2 | 1 | 3 | 10 |
| Berndt/2007 | 1 | 1 | 0 | 1 | 0 | 2 | 1 | 2 | 8 |
| Onsory/2008 | 1 | 1 | 0 | 0 | 0 | 2 | 0 | 1 | 5 |
| Beuten/2009 | 1 | 1 | 1 | 1 | 1 | 2 | 1 | 2 | 10 |
| Gupta/2010 | 1 | 1 | 1 | 0 | 1 | 0 | 0 | 2 | 6 |
| Sonada/2010 | 1 | 2 | 1 | 1 | 1 | 2 | 0 | 2 | 10 |
| Sissung/2010 | 1 | 2 | 1 | 1 | 1 | 2 | 1 | 2 | 11 |
| Szendroi/2011 | 1 | 1 | 1 | 1 | 1 | 2 | 1 | 2 | 10 |
| Safarinejad/2012 | 1 | 1 | 1 | 1 | 0 | 2 | 1 | 2 | 9 |
| Jurecekova//2013 | 1 | 1 | 1 | 1 | 1 | 2 | 0 | 3 | 10 |
| Suzuki/2003 | 1 | 1 | 1 | 0 | 0 | 0 | 0 | 2 | 5 |
| Balistreri 2011 | 1 | 1 | 0 | 0 | 1 | 0 | 1 | 1 | 5 |
| Chae/2009 | 1 | 2 | 1 | 0 | 1 | 2 | 1 | 3 | 11 |
| Lu/2015 | 1 | 1 | 1 | 1 | 0 | 2 | 1 | 3 | 10 |
| Chen/2007 | 1 | 2 | 0 | 0 | 0 | 2 | 0 | 3 | 8 |
| Nicolaiew/2009 | 1 | 1 | 1 | 1 | 0 | 2 | 0 | 1 | 7 |
| Robles-Fernandez/2017 | 1 | 1 | 1 | 1 | 1 | 2 | 0 | 2 | 9 |
| Tang/2018 | 1 | 1 | 1 | 1 | 1 | 2 | 1 | 3 | 11 |
|  |  |  |  |  |  |  |  |  |  |
